# Physics-Informed Machine Learning for Optimized and Sustainable Biochar Water Treatment

**DOI:** 10.3390/molecules31132349

**Published:** 2026-07-03

**Authors:** Qingyang Liu, Bing Bai

**Affiliations:** 1 Co-Innovation Center for the Sustainable Forestry in Southern China, College of Ecology and Environment, Nanjing Forestry University, Nanjing 210037, China; 2 Institute of Resources and Environment, Beijing Academy of Science and Technology, Beijing 100089, China; baibing_1029@163.com

**Keywords:** biochar, wastewater treatment, physics-informed machine learning, sustainability assessment, life cycle assessment

## Abstract

Biochar water treatment stands at a decisive crossroads, where the promise of large-scale application meets the reality of laboratory trial-and-error. This study contends that the fundamental bottleneck to progress lies in the field’s persistent reliance on empirical experimentation and black-box data models. We therefore propose a conceptual research paradigm that aims to deeply integrate physics-informed machine learning (PIML) with life cycle assessment (LCA). The novelty of this framework lies in three dimensions: (i) the bidirectional information flow between PIML and LCA, enabling simultaneous material design and sustainability assessment; (ii) the embedding of fundamental physical laws (adsorption isotherms, kinetics, thermodynamics) directly into learning architectures to ensure physical consistency; and (iii) the extension to a water–energy–soil–food closed-loop system for holistic resource management. While the individual components of this framework have been demonstrated in other domains, their integrated application to biochar water treatment remains in early development stages. This perspective outlines potential pathways and identifies critical research gaps that must be addressed to realize this vision. The focus is on charting future directions rather than reporting established achievements. Through critical evaluation, we assess current integrated models under small-sample constraints and explicitly pinpoint explainability and cross-scale generalization as two indispensable gaps that industrial deployment demands be bridged. Building on this foundation, we outline a blueprint for a closed-loop system coupling water, energy, soil, and food, and present a three-phase roadmap for future research. This study seeks to offer a constructive perspective with the hope of supporting biochar technology toward more sustainable implementation.

## 1. Introduction

Biochar is a carbon-rich, porous solid material produced through thermochemical conversion processes such as pyrolysis and hydrothermal carbonization of biomass feedstocks. These feedstocks include agricultural and forestry residues, livestock manure, and municipal sludge, and the processes are carried out under conditions of limited oxygen and low temperature [1]. Consequently, biochar has become a research hotspot in the fields of environmental functional materials and water treatment technologies. Biochar offers key advantages, including wide availability of raw materials, low production cost, eco-friendliness, a well-developed pore structure, a large specific surface area, and abundant surface functional groups [2]. Owing to these advantages, biochar demonstrates significant application potential in water pollution control and aquatic ecosystem restoration. Studies have confirmed that unmodified raw biochar possesses a certain capacity for removing heavy metal ions, organic pollutants, and nutrients from water. However, when physically activated, chemically modified, or engineered into composites, biochar-based materials can achieve performance improvements by orders of magnitude [3].

Currently, the development and application of biochar-based water treatment materials rely heavily on the traditional laboratory trial-and-error research model, which represents a significant challenge hindering the transition from lab-scale to industrial and large-scale deployment [4]. At the material design level, there exists a highly complex nonlinear coupling relationship among preparation parameters (e.g., pyrolysis temperature, feedstock ratio, modifier dosage), microstructure, and macroscopic performance. Traditional methods can optimize only one or two variables at a time, making it impossible to systematically explore the entire parameter space and thus easily missing optimal synthesis conditions. At the mechanistic understanding level, many studies rely primarily on characterization-based inference, with limited quantitative modeling, which further exacerbates the randomness in material design and process optimization. At the engineering application level, laboratory studies typically employ idealized conditions such as single contaminants, deionized water systems, constant temperature and pH, and static batch reactions. These conditions differ significantly from real industrial wastewater (e.g., coexisting pollutants, high salinity, high hardness, fluctuating water quality, and dynamic continuous flow), rendering the optimal conditions identified in the laboratory difficult to transfer directly to field applications [5]. These combined limitations contribute to the well-recognized industry challenge in which many laboratory-scale successes face substantial barriers to real-world translation.

However, most existing machine learning models in biochar-based water treatment are purely data-driven black-box models that rely on statistical correlations from experimental data for learning and prediction, without incorporating any fundamental principles or theoretical constraints from physics, chemistry, or environmental engineering [6]. This leads to several limitations that require attention. First, a key limitation is the lack of physical consistency in predictions. In regions beyond the training data, especially sparse or extrapolation areas, models frequently generate results that contradict fundamental scientific principles, including mass conservation, reaction kinetics, and adsorption thermodynamics, which ultimately results in poor reliability. Second, a major issue is the poor interpretability of current models. Existing explanation tools like SHAP, partial dependence plot, and local interpretable model-agnostic explanations are limited to analyzing statistical correlations between input features and output outcomes [7]. As a result, they cannot uncover the causal mechanisms that connect preparation parameters to structural evolution and ultimately to performance changes, which severely hampers guided material design. Third, generalization across different scenarios remains weak; models trained on a specific feedstock or class of pollutants cannot be directly transferred to other systems, limiting their adaptability and universality. Fourth, these models perform poorly with small sample sizes. Biochar experimental datasets typically have small sample volumes, high dimensionality, significant noise, and low standardization, making pure data-driven models prone to overfitting and severely degrading generalization performance [8]. It is important to acknowledge that recent advances in hybrid machine learning and interpretable artificial intelligence have made notable progress in addressing some of these limitations. For instance, ensemble learning methods combined with SHAP analysis have improved feature interpretability in adsorption prediction tasks, while hybrid models integrating Gaussian process regression with mechanistic knowledge have shown promise for small-sample environmental applications. Additionally, transfer learning and meta-learning approaches have been explored to leverage knowledge across different biochar systems. However, these approaches still primarily rely on statistical correlations and lack the rigorous enforcement of physical laws. PIML extends these efforts by embedding fundamental conservation principles and kinetic equations directly into the learning process, thereby offering a more principled pathway to achieving physical consistency and extrapolative reliability [9].

In contrast, PIML emerges as a new generation of artificial intelligence paradigm, which incorporates prior knowledge such as physical conservation laws, constitutive equations, kinetic rules, and thermodynamic principles into model architecture, loss functions, and training processes [10]. As a result, it achieves deep integration of data-driven approaches with physical constraints. This paradigm retains the strengths of machine learning in handling complex nonlinear problems while ensuring scientific validity and rationality through the enforcement of physical laws. It effectively addresses key challenges such as small sample sizes, extrapolation prediction, and poor interpretability, offering a novel pathway toward the intelligent advancement of biochar-based water treatment technologies. Concrete examples of embeddable physical knowledge include adsorption isotherms like the Langmuir equation, which describes monolayer adsorption equilibrium [11]. Further examples are adsorption kinetics, such as the pseudo-second-order model that governs the rate of adsorption, and mass conservation principles for batch systems [12]. In catalytic degradation processes, the Arrhenius equation can constrain temperature-dependent reaction rates, while electron transfer rate equations can describe non-radical pathways. These equations can be incorporated as either hard constraint embedded within neural network architectures or soft constraints added to loss functions, ensuring that predictions remain physically consistent across diverse operating conditions.

To overcome the significant challenge and technical shortcomings in the biochar water treatment field, this study proposes a transformative research framework. This framework consists of an efficient and sustainable biochar water treatment system that deeply couples PIML with LCA. This paradigm aims to move beyond the limitations of traditional trial-and-error methods and purely data-driven black-box models by adopting a strategy centered on physics-based constraints, data-driven learning, and lifecycle sustainability. The focus shifts from passive performance prediction to active system optimization and targeted sustainable design, establishing an end-to-end technical framework that integrates materials design, process optimization, environmental assessment, economic evaluation, and system integration. To support the critical perspective presented, a structured literature search was conducted using Web of Science, Scopus, and Google Scholar, covering publications from 2015 to 2025. Keywords included combinations of: “biochar water treatment”, “machine learning”, “physics-informed machine learning”, “adsorption prediction”, and “life cycle assessment”. The search initially identified 847 records across the three databases. After removing duplicates (n = 213), 634 records underwent title and abstract screening. Studies were included if they met the following criteria: (1) focused on biochar for water treatment applications; (2) employed machine learning or statistical modeling approaches; (3) addressed sustainability assessment or life cycle considerations; or (4) discussed integrated modeling frameworks. Exclusion criteria were: conference abstracts (n = 89), non-English publications (n = 67), studies lacking empirical or simulation data (n = 128), and studies not directly relevant to biochar water treatment applications (n = 207). This resulted in 143 full-text articles for detailed review, from which 74 key references were selected to support the critical analysis presented in this perspective. This systematic approach ensures comprehensive coverage of the relevant literature while maintaining focus on the core themes of the manuscript.

**Figure 1:**
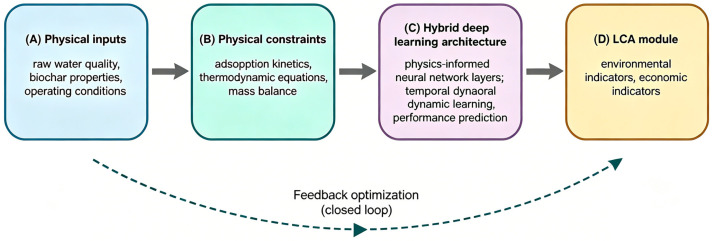
Schematic framework of PIML for biochar water treatment systems with integrated LCA and multi-objective optimization. The framework consists of four interconnected modules. Module A defines the physical inputs, including raw water quality parameters (pH, temperature, pollutant concentration, coexisting ions), biochar properties (specific surface area, pore volume, functional group content), and operating conditions (adsorbent dosage, reaction time, regeneration cycles). Module B establishes the physical constraints embedded in the learning process, covering adsorption isotherms (Langmuir, Freundlich, Sips), kinetic models (pseudo-first-order, pseudo-second-order, intraparticle diffusion), thermodynamic relationships (Gibbs free energy, enthalpy-entropy compensation), and mass conservation equations. Module C presents the hybrid PIML architecture, a deep learning framework that integrates data-driven neural network layers with physics-informed residual-based regularization. Module D comprises the LCA and optimization component, which translates PIML outputs into environmental metrics and economic indicators. Solid arrows denote forward prediction, while dashed arrows represent feedback optimization loops.

The novelty of the proposed PIML-LCA integration lies in bidirectional information flow. Unlike conventional approaches where LCA is conducted posthoc based on experimental results, our framework enables LCA-guided PIML model training, where environmental impact indicators serve as additional optimization objectives. Specifically, PIML predicts performance metrics (e.g., adsorption capacity, degradation rate, regeneration cycles) under various material and operational conditions; these outputs feed directly into the LCA module, which calculates life-cycle environmental footprints (e.g., global warming potential, energy consumption). The combined performance-environmental indicators are then used as multi-objective optimization targets, enabling simultaneous material design and sustainability assessment. By jointly optimizing treatment efficiency and environmental performance, this closed-loop integration enables rapid biochar screening that is unattainable through traditional stepwise processes. A conceptual workflow (Figure 1) illustrates the information flow from physical constraints through PIML predictions to LCA-based sustainability evaluation and multi-objective optimization. It is important to emphasize that the framework proposed herein represents a forward-looking perspective rather than a validated methodology. While PIML has demonstrated success in fluid dynamics, materials science, and climate modeling, its application to biochar water treatment remains largely exploratory [10]. Similarly, LCA has been applied to biochar systems [6,11], but its integration with PIML for multi-objective optimization has not been implemented. Throughout this manuscript, we explicitly distinguish between current capabilities supported by published literature, emerging approaches under active development, and long-term visions requiring substantial future research.

## 2. Current Status

### 2.1. Connecting Biochar Preparation, Structure, and Performance

#### 2.1.1. Preparation and Modification Methods

The physicochemical structure and application performance of biochar are determined by its preparation methods and modification strategies. Currently, biochar production has evolved into a technical system centered on pyrolysis, supplemented by hydrothermal carbonization, with multiple thermochemical processes coexisting [1,12]. Pyrolysis is the most widely used method and can be classified based on heating rate, residence time, and reaction temperature into fast pyrolysis (300–1000 °C, residence time <2 s), slow pyrolysis (100–1000 °C, residence time 5–30 min), and medium-speed pyrolysis. Slow pyrolysis yields high biochar yields (45–70%) and produces stable pore structures, making it the dominant process for biochar tailored to water treatment applications [6]. Hydrothermal carbonization is suitable for high-moisture feedstocks such as sludge and livestock manure, converting them at temperatures of 180–260 °C under autogenous pressure (2–5 MPa). The resulting biochar features abundant surface functional groups but relatively low porosity. Additionally, emerging techniques such as microwave carbonization, gasification, and flash carbonization offer advantages including uniform heating and high reaction efficiency, and are gradually entering the exploratory stages of practical application [2].

Among preparation parameters, pyrolysis temperature is the key variable controlling biochar structure. Low-temperature pyrolysis (≤400 °C) retains numerous oxygen-containing functional groups (hydroxyl and carboxyl groups), giving the biochar strong hydrophilicity and enabling effective adsorption of polar heavy metal ions and polar organic compounds via hydrogen bonding and electrostatic interactions [2]. Moderate-temperature pyrolysis (400–600 °C) promotes gradual pore development, increasing specific surface area and significantly enhancing the number of adsorption sites [13]. High-temperature pyrolysis (>600 °C) increases graphitization and aromaticity, maximizing specific surface area and pore volume; in this case, π–π interactions and hydrophobic effects become dominant, leading to excellent adsorption capacity toward hydrophobic organic pollutants such as antibiotics, dyes, and polycyclic aromatic hydrocarbons [3].

To overcome the performance limitations of pristine biochar, researchers have developed five major modification strategies to comprehensively enhance water treatment capabilities of biochar [5]. The first strategy is physical/chemical activation, where physical activation uses gases such as CO_2_, steam, or NH_3_ to etch pores, expanding pore volume and increasing specific surface area, while chemical activation involves impregnating biochar with acid (e.g., HCl, H_2_SO_4_), alkali (e.g., NaOH, KOH), or salt (e.g., ZnCl_2_) solutions to introduce oxygen- and nitrogen-containing functional groups, thereby optimizing pore structure. The second strategy is non-metallic heteroatom doping, which incorporates heteroatoms such as N, S, P, and B to alter the charge distribution and electronic structure of the carbon framework, creating defect-based active sites that improve adsorption selectivity and catalytic activity; nitrogen doping, in particular, forms active sites such as graphitic nitrogen, pyridinic nitrogen, and pyrrolic nitrogen, significantly enhancing electron transfer efficiency during advanced oxidation processes (AOPs) [14]. The third strategy is metal/metal oxide loading, where loading zero-valent iron, iron oxide, zinc oxide, or manganese dioxide nanoparticles onto biochar imparts magnetic properties to facilitate solid–liquid separation and catalytic activity to activate persulfate or hydrogen peroxide, while also increasing adsorption sites for heavy metals [15]. The fourth strategy is layered double hydroxides composite modification, which combines layered double hydroxides with biochar to leverage the interlayer anion exchange capacity of layered double hydroxides and their ability to reduce and immobilize heavy metals, along with porous support characteristics of biochar, resulting in significantly enhanced removal efficiencies for heavy metals, phosphate, and anionic pollutants [16,17]. The fifth strategy is organic functional group grafting, where functional molecules such as β-cyclodextrin, polyethyleneimine, or polyaniline are grafted to introduce abundant active sites including amino groups, hydroxyl groups, and cyclodextrin cavities [18,19].

#### 2.1.2. Removal Mechanisms

The removal of water pollutants by biochar primarily occurs through two major pathways: adsorption and catalytic degradation. The mechanisms vary depending on the type of pollutant, often involving synergistic effects of multiple processes [5]. Adsorption is the core mechanism for biochar to remove pollutants and applies to most contaminants, including heavy metals, antibiotics, dyes, and nutrients. The microporous and mesoporous structures of biochar physically trap pollutant molecules through pore-filling; this mechanism dominates at low concentrations, and adsorption capacity is highly correlated with pore volume and pore size distribution [1]. Polar functional groups (e.g., hydroxyl, carboxyl, and amino groups) on the biochar surface form hydrogen bonds with electronegative atoms (O, N, F) in pollutant molecules, playing a crucial role in adsorbing polar compounds. Electrostatic interactions depend on solution pH and the biochar’s point of zero charge (pH_PZC_): when pH < pH_PZC_, the positively charged surface adsorbs anionic pollutants; when pH > pH_PZC_, the negatively charged surface favors adsorption of cationic heavy metals and cationic dyes [20]. The hydrophobic aromatic structure of high-temperature biochar attracts nonpolar organic pollutants via hydrophobic interactions, enabling efficient enrichment. Aromatic ring structures in biochar interact with aromatic rings in pollutants such as antibiotics, dyes, and polycyclic aromatic hydrocarbons through π–π conjugated stacking, serving as the dominant mechanism for adsorbing aromatic organic pollutants [3,21]. Additionally, functional groups on the biochar surface or immobilized metal sites form surface complexes with heavy metal ions or exchange with ions in water, effectively immobilizing heavy metals through ion exchange or surface complexation [22].

For layered double hydroxides composites, additional mechanisms such as reduction, isomorphous substitution, and surface precipitation contribute to heavy metal removal. For example, Cr(VI) can be reduced to less toxic Cr(III) by reductive groups on the biochar surface, followed by isomorphous substitution with metal ions in layered double hydroxides, while Pb^2+^, Cd^2+^, and other heavy metals may precipitate as carbonates or hydroxides on the material surface, achieving stable immobilization [17,23].

Catalytic degradation mainly occurs during AOPs involving biochar and targets refractory organic pollutants. This process can be categorized into radical and non-radical pathways [24]. In the radical pathway, biochar acts as a catalyst or support to activate oxidants such as persulfate, hydrogen peroxide, or ozone, generating highly reactive species like hydroxyl radicals (•OH), sulfate radicals (SO_4_^−^•), and superoxide radicals (O_2_^−^•). These species non-selectively oxidize and decompose organic pollutants, offering fast reaction rates and high mineralization efficiency [25]. In contrast, the non-radical pathway is centered on singlet oxygen (^1^O_2_) and direct electron transfer, featuring strong selectivity and minimal interference from water matrix components such as coexisting ions and natural organic matter, thus providing greater stability [26]. In the electron-transfer pathway, biochar serves as an electron shuttle, facilitating electron transfer between pollutants and oxidants and enabling selective degradation [27].

### 2.2. Data-Driven Methods for Biochar Water Treatment

#### 2.2.1. Common Machine Learning Algorithms and Applications

Machine learning has become a vital tool in biochar-based water treatment research due to its ability to handle nonlinear, multivariate, and high-dimensional data [6]. Different algorithms exhibit significant variations in suitability and performance. The major algorithms and their applications are summarized in Table 1.

In practical applications, machine learning models primarily fulfill three functions. The first is predicting material properties. This involves estimating biochar-specific surface area, pore volume, functional group content, adsorption capacity, and catalytic activity based on biomass feedstock, pyrolysis parameters, and modification conditions [6]. The second is optimizing process parameters. Machine learning identifies optimal operational conditions such as pH, dosage, reaction time, initial concentration, and temperature [32]. The third is supporting mechanistic analysis. By using feature importance ranking, machine learning models identify critical parameters that affect biochar performance, thereby guiding mechanistic research [7].

#### 2.2.2. Dominant Factors Identified

Using interpretability tools such as SHapley Additive explanations (SHAP), partial dependence plots, and feature importance scores, existing machine learning studies have identified four major categories of key features that govern biochar water treatment performance [34]. These contribution percentages were derived from meta-analyses of multiple machine learning studies in which feature importance was calculated using SHAP values and permutation feature importance scores. Specifically, the percentage contribution of each feature category was computed as the average SHAP value across all features within that category, normalized by the sum of average SHAP values across all categories. The ranges reflect variations across different pollutant systems and biochar types reported in the literature. Preparation condition features (e.g., pyrolysis temperature, time, heating rate, modifier dosage) account for 3% to 65% of the contribution, with pyrolysis temperature being the primary factor affecting biochar yield, specific surface area, graphitization degree, and functional group types [8]. Material intrinsic features (e.g., specific surface area, pore volume, pore size distribution, carbon content, heteroatom doping level, functional groups) contribute 20% to 35% and serve as core internal determinants of adsorption capacity and catalytic activity [6]. Water treatment operating conditions represent the most influential category, with solution pH consistently recognized as the primary operational parameter, contributing up to 41% to model predictions; other significant factors include initial pollutant concentration, biochar dosage, reaction time, and temperature [32]. Water quality environmental features (coexisting ions including Ca^2+^, Mg^2+^, Cl^−^, CO_3_^2−^, natural organic matter, ionic strength, water temperature) affect treatment efficiency through competitive adsorption, active site occupation, and radical quenching mechanisms [5]. However, due to the lack of physical constraints in purely data-driven models, these analyses cannot reveal the coupling mechanisms or underlying physical laws among features, thus limiting the reliability of optimization outcomes.

### 2.3. From Black Box to Physics-Informed Frameworks

While Section 2.2.2 demonstrated how existing machine learning studies have identified key features influencing biochar performance through statistical interpretability tools, these approaches remain limited to correlation-based analyses. The transition to physics-informed models addresses fundamental limitations that purely data-driven approaches cannot overcome, regardless of the sophistication of interpretability tools. This section analyzes the critical shortcomings of black-box models and articulates why incorporating physical knowledge is essential for achieving reliable, interpretable, and generalizable biochar water treatment models. Although purely data-driven machine learning models have achieved incremental progress in biochar-based water treatment research, their inherent limitations prevent them from supporting industrial-scale and sustainable development. Therefore, transitioning toward physics-informed models is desirable and increasingly necessary [9].

Physical constraints are essential for model reliability. Pure data models only learn statistical correlations between variables without adhering to physical laws such as adsorption thermodynamics, reaction kinetics, and mass conservation [35]. In parameter ranges not covered by training data, for example, ultra-high pyrolysis temperatures, extremely low pH values, or high pollutant concentrations, these models may produce unphysical results. Such results include adsorption capacities exceeding theoretical limits or reaction rates violating kinetic principles, rendering these models unsuitable for engineering extrapolation. In contrast, PIML incorporates physical equations directly into the model. This approach enforces outputs that conform to natural laws and ensures fundamental reliability in predictions [10].

Furthermore, interpretability is a prerequisite for targeted materials design. The core objective of biochar water treatment technology is to achieve targeted design and on-demand preparation. This means precisely designing the structure and performance of biochar based on specific pollutants and water quality conditions. Pure black-box models cannot explain why a certain parameter improves performance, nor can they clarify the causal relationship between structure and performance. They only provide statistical correlations. In contrast, physics-informed models are grounded in physical mechanisms. They explicitly analyze the causal chain that connects preparation parameters to microstructure and then to performance output. As a result, they offer theoretical guidance for targeted material modification [7].

Moreover, adaptability under small-sample conditions represents a practical necessity in this field. Biochar experiments are time-consuming and costly, making it difficult to obtain large-scale standardized datasets. Existing data commonly suffer from small sample sizes, high dimensionality, significant noise, and low standardization. Pure data-driven models are highly prone to overfitting and exhibit poor generalization under small-sample conditions [8]. Physics-informed models, by incorporating prior physical knowledge, significantly reduce reliance on large datasets, enabling efficient learning and accurate prediction even with limited data [10]. In summary, transitioning from pure data-based black-box models to physics-informed integrated models is a promising pathway toward breaking through development challenges and achieving intelligent, scalable engineering solutions in biochar water treatment technology.

Using interpretability tools such as SHAP, partial dependence plots, and feature importance scores, existing machine learning studies have identified four major categories of key features that govern biochar water treatment performance, each with clear contributions and influence patterns [34]. Preparation condition features account for 3% to 65% of the contribution, with pyrolysis temperature being the primary factor affecting biochar yield, specific surface area, graphitization degree, and functional group types, while pyrolysis time, heating rate, and modifier dosage serve as secondary factors [34]. Material intrinsic features contribute 20% to 35% and include specific surface area, total pore volume, pore size distribution, carbon content, heteroatom doping level, and the types and contents of functional groups, all of which are core internal determinants of adsorption capacity and catalytic activity. Water treatment operating conditions represent the most influential category, with a contribution as high as 41%. Solution pH is consistently recognized across all models as the primary operational parameter, directly controlling the surface charge of biochar and pollutant speciation, while other significant factors include initial pollutant concentration, biochar dosage, reaction time, and temperature. Finally, water quality environmental features such as coexisting ions including Ca^2+^, Mg^2+^, Cl^−^, and CO_3_^2−^, natural organic matter, ionic strength, and water temperature affect treatment efficiency through competitive adsorption, occupation of active sites, and radical quenching mechanisms. The above feature importance analysis provides a data-driven foundation for preliminary optimization in biochar preparation and water treatment processes. However, due to the lack of physical constraints in purely data-driven models, they cannot reveal the coupling mechanisms or underlying physical laws among features, thus limiting the reliability of optimization outcomes [34].

## 3. Key Challenges

### 3.1. Model Robustness and Interpretability Under Small-Sample Conditions

#### 3.1.1. Characteristics and Challenges of Biochar Data

Experimental data in the biochar water treatment field exhibit five typical characteristics: small sample size, high dimensionality, strong noise, lack of standardization, and sparsity [8]. These characteristics pose major challenges for machine learning applications. Sample sizes are extremely limited. A single research paper typically generates only dozens to hundreds of valid data points, and large-scale publicly available datasets are scarce, failing to meet the training requirements of conventional deep learning models. In addition, features are highly dimensional and strongly coupled. Influencing factors span four categories, namely preparation, modification, operation, and water quality, comprising dozens of variables. Many of these variables exhibit complex nonlinear interactions, such as synergistic effects between pyrolysis temperature and heteroatom doping levels, making individual feature interpretation difficult [4]. Furthermore, data contain substantial noise and bias. Differences in characterization equipment, testing methods, and experimental procedures across laboratories lead to significant deviations in data under identical conditions, while experimental and measurement errors further amplify noise [35,36]. Another issue is the lack of data standardization. There are no unified standards for raw material descriptions, preparation parameters, performance testing methods, or characterization metrics, preventing cross-study data integration. Finally, the parameter space is highly sparse. Experimental parameters cover only limited ranges, leaving numerous critical combinations unexplored, resulting in vast gaps in the data space and making model extrapolation extremely difficult. These data characteristics result in poor robustness, severe overfitting, and weak generalization capabilities in traditional machine learning models, rendering them unsuitable for real-world applications [8].

#### 3.1.2. Improving Model Reliability Through Physical Constraints

PIML enhances model reliability and robustness under small-sample conditions by embedding physical laws into models at three levels: architecture, loss function, and training process [10]. Hard constraints are applied by integrating physical equations directly into the neural network layers or computational units of machine learning models. These equations include adsorption isotherm models such as Langmuir, Freundlich, and Sips [11], adsorption kinetics models such as pseudo-first-order, pseudo-second-order, and intraparticle diffusion [12,37], as well as advanced oxidation reaction kinetics and mass conservation laws. This forces outputs to conform to physical principles and eliminates unphysical predictions. Soft constraints are implemented via penalty terms. Physical violation penalties are added to the model loss function. When predictions deviate from physical laws, the loss value increases automatically, guiding the model toward physically consistent optimization. This approach is particularly useful for complex processes where direct equation integration is impractical. Physics-based data augmentation is also employed. Synthetic data, or virtual samples, are generated using known physical models to expand the training dataset, effectively addressing the limitations posed by small sample sizes [9]. A hybrid strategy combining physics-based model prediction with machine learning correction is adopted. In this strategy, the physics-based model provides the underlying trend and machine learning fits the residuals, thus balancing physical accuracy and data fitting capability. After optimization with physical constraints, preliminary studies in adjacent environmental modeling domains suggest that models may reduce prediction errors substantially in scenarios with small sample sizes and sparse data, thereby potentially enhancing robustness and reliability. However, it is important to note that these performance improvements have been demonstrated primarily in other fields such as fluid dynamics and climate modeling, and direct biochar-specific validation remains limited [10]. We therefore emphasize that these results should be interpreted as promising indicators rather than established benchmarks for biochar applications.

#### 3.1.3. Applicability of Explainability Tools

Traditional statistical interpretability tools, including SHAP, partial dependence plots, and LIME, can provide correlation-based explanations between features and outputs [7]. However, these tools are fundamentally limited to describing statistical associations and cannot uncover causal mechanisms connecting preparation parameters to structural evolution and ultimately to performance changes. Within a PIML framework, a dual-dimensional explainability system integrating physics and statistics must be established to achieve not only descriptive understanding but also causal explanation. This system comprises three layers of tools: (1) physical consistency verification tools that validate whether model predictions align with adsorption thermodynamics, kinetics, and mass transfer principles; (2) sensitivity analysis tools that quantify the influence of individual physical parameters on predictions, identifying which mechanisms dominate performance under specific conditions; and (3) hybrid interpretability tools that combine physical model outputs with statistical feature importance to trace the causal chain from preparation conditions through structural characteristics to performance outcomes [9].

#### 3.1.4. Challenges and Limitations of PIML in Environmental Applications

Despite its theoretical promise, the practical application of physics-informed machine learning to biochar-based water treatment is constrained by several significant challenges that merit careful consideration. First, data quality and heterogeneity remain critical issues. Although PIML requires less data than purely data-driven machine learning, it still depends on sufficient high-quality information to train its empirical components. The existing biochar literature is marked by considerable inconsistency, including varied characterization techniques, non-uniform reporting practices, and divergent experimental protocols [36]. Such heterogeneity can introduce systematic biases that physical constraints alone are unable to correct. As a result, models may satisfy physical laws while still producing empirically inaccurate predictions. Second, the physical models embedded within PIML frameworks are themselves approximations. Classical adsorption isotherms, such as those proposed by Langmuir and Freundlich, were developed for simplified, idealized systems and may not adequately capture the complexity of real biochar surfaces, which often exhibit heterogeneous active sites, multi-layered adsorption behavior, and synergistic interactions among adsorbates [11]. When such approximate laws are imposed as hard constraints, they risk propagating and even magnifying model errors, particularly in extrapolation scenarios where these approximations are most questionable. Third, conflicting predictions can arise from different physical principles. For instance, thermodynamic considerations might favor enhanced adsorption at elevated temperatures, whereas kinetic arguments could suggest slower attainment of equilibrium under the same conditions. Reconciling these competing perspectives demands careful prioritization or the formulation of multi-objective physics loss functions, which adds considerable complexity to the modeling process. Fourth, computational expense poses a practical obstacle. Physics-informed neural network architectures, especially those designed to solve partial differential equations, are notoriously costly to train [10]. Simulating coupled adsorption–diffusion–reaction systems often involves computing high-order derivatives, necessitating substantial computational power and specialized numerical methods. Fifth, transferability across real-world environmental systems is far from guaranteed. Models trained under controlled laboratory conditions may struggle to perform reliably in complex natural water matrices, where factors such as multiple coexisting pollutants, variable ionic strength, and dissolved organic matter can profoundly alter system behavior [5]. While the underlying physical mechanisms remain unchanged, the parameterization of these mechanisms including adsorption equilibrium constants and mass transfer coefficients can vary widely across different environmental contexts. Finally, the scarcity of industrial-scale operational data presents an even more fundamental limitation than the lack of laboratory measurements. The transfer learning and active learning strategies discussed earlier presuppose at least some availability of field or plant-level data, yet such information is often treated as proprietary or is simply not accessible, severely constraining the practical deployability of PIML approaches [38]. These limitations do not invalidate the PIML approach but underscore that careful problem formulation, appropriate architecture selection, and realistic expectations regarding model capabilities are essential. We recommend that future research prioritize benchmarking PIML against conventional ML approaches across diverse biochar systems to quantify the practical value added by physics constraints.

### 3.2. Cross-Scale Generalization Capability

#### 3.2.1. Nonlinear Mapping Challenge from Laboratory to Industrial Scale

The engineering application of biochar-based water treatment technologies requires a cross-scale transition from laboratory-scale bench tests through pilot-scale trials to full industrial scale. However, significant differences between laboratory and industrial settings create a severe nonlinear mapping challenge [4]. Laboratory conditions are ideal—static batch reactions, single target pollutants, deionized water systems, constant temperature and pH, absence of interfering ions, low dosing levels, and short reaction times. In contrast, industrial settings involve complex operating conditions: dynamic continuous-flow reactors, coexistence of multiple pollutants causing competitive adsorption or radical quenching, high-salinity, high-hardness, or high-organic-matter water quality, substantial diurnal and seasonal fluctuations in water quality, large-scale reactors, long-term continuous operation, packing material clogging, and performance degradation [5]. Traditional models trained solely on laboratory data often fail to account for scale effects, mass transfer effects, and matrix interference effects [39].

#### 3.2.2. Potential Solutions Through Transfer Learning and Domain Adaptation

To bridge the laboratory-to-industrial gap, PIML can be integrated with three complementary strategies that enable efficient knowledge transfer with limited industrial data. Transfer learning preserves the physical constraint layer and feature extraction layers from laboratory-trained source models, requiring only a small amount of industrial field data to fine-tune top-level parameters [38]. Domain adaptation aligns feature distributions between source and target domains through distribution-matching algorithms, allowing the model to learn universal physical laws rather than scenario-specific patterns. Active learning strategically selects the most informative industrial samples for experimental testing, maximizing performance improvement at minimal cost [40].

The theoretical potential of transfer learning, domain adaptation, and active learning for reducing cross-scale prediction errors is substantial, as demonstrated in adjacent fields such as climate modeling and biomedical engineering [38]. However, direct evidence from biochar water treatment applications is currently limited, as comprehensive industrial datasets enabling rigorous validation remain scarce. We hypothesize that with sufficient industrial monitoring data, cross-scale prediction errors could be progressively reduced toward engineering-acceptable levels. Achieving this target requires: (i) investment in industrial monitoring and data collection infrastructure; (ii) development of robust domain adaptation algorithms; (iii) active learning campaigns that strategically sample the most informative industrial conditions; and (iv) benchmarking studies comparing PIML-based transfer learning against conventional approaches. This represents a critical research frontier rather than an established capability.

In summary, the transition from purely data-driven black-box models to physics-informed integrated models represents a promising pathway toward overcoming the development challenges and achieving intelligent, scalable engineering solutions in biochar water treatment technology. The following section presents a detailed framework for implementing this transition, addressing the specific challenges of model robustness, interpretability, and cross-scale generalization through the systematic integration of physical knowledge and sustainability assessment.

## 4. A Closed-Loop Paradigm

### 4.1. Framework Construction of PIML

#### 4.1.1. Types of Embeddable Physical Knowledge

For adsorption-related processes, the key physical knowledge includes adsorption isotherms, kinetics, and thermodynamics. Isotherm models (Langmuir, Freundlich, Sips, Temkin) describe equilibrium relationships between adsorbate concentration and adsorption capacity [11]. Kinetic models (pseudo-first-order, pseudo-second-order, intraparticle diffusion, liquid film diffusion) characterize rate-controlling steps and temporal evolution [12,37]. Thermodynamic relationships (Gibbs free energy, enthalpy, entropy, Van’t Hoff equation) enable prediction of adsorption spontaneity and temperature dependence [41].

For catalytic degradation processes, physical knowledge encompasses radical and non-radical pathways, along with mineralization kinetics. Radical pathways involve reaction networks for reactive species (•OH, SO_4_^−^•, O_2_^−^•) and the Arrhenius relationship linking rate constants to temperature. Non-radical pathways include singlet oxygen (^1^O_2_) generation mechanisms, electron transfer rate equations, and conductivity-correlated efficiency [26]. Mineralization kinetics relate total organic carbon removal to reaction time [25].

For mass transfer and reactor engineering, physical knowledge includes mass transfer mechanisms, reactor models, and scale-up effects. Mass transfer is described by membrane diffusion, intraparticle diffusion, and fixed-bed coefficient models [39]. Reactor models encompass fixed-bed breakthrough, continuous stirred-tank, and plug-flow reactor formulations for different flow regimes [42]. Scale-up correction models account for differences in mass transfer and fluid dynamics between laboratory and industrial scales [43].

#### 4.1.2. Multi-Task Learning Framework

To address the small-sample and generalization challenges identified in Section 3, a multi-task PIML framework is developed, enabling a single model to simultaneously learn and predict multiple related tasks. This framework comprises four predictive task categories that correspond to key aspects of biochar water treatment systems.

The first task predicts biochar intrinsic properties, including specific surface area, pore volume, graphitization degree, and functional group content [6]. The second task predicts pollutant removal performance, covering adsorption capacity, degradation rate, mineralization rate, and removal efficiency [5]. The third task predicts water quality anti-interference capability, quantified through influence coefficients of coexisting ions and natural organic matter [34]. The fourth task predicts cycling stability performance, encompassing regeneration cycles, performance decay rate, and service life [4].

By leveraging shared low-level physical feature extraction layers across all four tasks, this multi-task framework exploits inherent correlations among the predictive objectives. For instance, biochar pore structure (Task 1) directly influences adsorption capacity (Task 2), while surface functional groups (Task 1) affect both anti-interference capability (Task 3) and stability during regeneration (Task 4). This shared representation learning significantly improves sample efficiency and prediction accuracy under small-sample conditions compared to training separate single-task models [9].

#### 4.1.3. PIML Architectures and Implementation Strategies

The practical implementation of PIML for biochar water treatment can follow several architectural paradigms, each with distinct advantages and trade-offs. However, it is important to acknowledge that these approaches carry significant computational costs. Physics-informed neural network architectures, especially those designed to solve partial differential equations, are notoriously costly to train [10]. Simulating coupled adsorption–diffusion–reaction systems often involves computing high-order derivatives, necessitating substantial computational power and specialized numerical methods. The computational expense scales with problem dimensionality and the complexity of embedded physics, potentially limiting practical application in resource-constrained settings. Furthermore, the choice of which physical constraints to embed presents a non-trivial decision. Embedding overly simplified models (e.g., Langmuir isotherm for heterogeneous surfaces) may introduce systematic biases, while embedding highly complex models may render training infeasible [11]. Careful problem formulation, appropriate architecture selection, and realistic expectations regarding model capabilities are therefore essential:

(i) Physics-Informed Neural Networks: The most widely adopted PIML architecture extends standard neural networks by incorporating physical constraints directly into the loss function [10]. For a generic forward problem, the loss function takes the form
  (1) $$L\left(\theta \right)=L_{data}\left(\theta \right)+\lambda_{phy}L_{phy}(\theta )$$
  where
  (2) Ldataθ=1N∑i=1N∣∣y^i−yi∣∣2
  is the data mismatch term, and
  (3) $$L_{phy}\left(\theta \right)=\frac{1}{M}\sum_{J=1}^{M}{||F\left(x_{j};\theta \right)||}^{2}$$
  is the physics residual term enforcing the governing equation F(x) = 0.The hyperparameter λ_phy_ balances data fidelity against physical consistency. For adsorption prediction, F(t) could represent the residual of the pseudo-second-order kinetic equation [12]:
  (4) Ft=dqtdt−k2(qe−qt)2.
(ii) Physics-constrained ensemble learning: For cases where direct embedding of partial differential equations is challenging, physics constraints can be imposed through ensemble architectures. Multiple base learners (e.g., random forest, gradient boosting, support vector machines) are trained, and their predictions are weighted using a physics-based consistency score. This approach offers greater flexibility but weaker physical guarantees compared to PINNs [9].
(iii) Hybrid physics-ML correction models: A mechanistic model provides baseline predictions, and the machine learning component learns the residual between the mechanistic model and observed data:
  (5) y^ =MPhyx+MMLx
  This approach is particularly suitable when physical models are well-established but imperfect, as is often the case with adsorption isotherm models that assume idealized conditions [11].
(iv) Bayesian physics-informed learning: For uncertainty quantification, Bayesian neural networks can be combined with physical constraints. The posterior distribution over network parameters incorporates physical knowledge through the prior distribution, and predictions are expressed as posterior predictive distributions, enabling confidence interval estimation [10].

#### 4.1.4. Validation Procedures and Uncertainty Quantification

Rigorous validation of physics-informed machine learning models for biochar water treatment demands a multifaceted strategy that goes beyond conventional performance metrics [9]. One essential dimension is physical consistency testing, wherein model outputs are systematically checked against the fundamental principles they are intended to honor. For adsorption systems, this entails verifying thermodynamic feasibility, such as confirming negative Gibbs free energy changes for spontaneous processes, ensuring that kinetic rate constants fall within physically plausible ranges, and confirming strict mass balance closure [41]. A practical way to operationalize this is through a physical consistency score, calculated as the proportion of test instances that simultaneously satisfy all imposed physical constraints. Another critical component is the assessment of extrapolation performance. Standard train–test splits are insufficient for revealing a model’s true generalization capacity. Instead, evaluation should extend to parameter regimes that lie outside the training distribution—for example, predicting adsorption behavior at temperatures or pH levels not represented in the training set. Such testing provides a direct measure of how effectively the model harnesses embedded physical knowledge to compensate for limited data coverage [10]. Equally important is uncertainty quantification, which should be decomposed into its two constituent sources: aleatoric uncertainty, arising from inherent noise in the data, and epistemic uncertainty, stemming from incomplete knowledge of model parameters. Bayesian formulations of PIML naturally offer this decomposition, while ensemble-based methods can approximate predictive variance by examining the spread across multiple independently trained models [44]. For any prediction intended to inform decision-making, accompanying confidence intervals are indispensable. Sensitivity analysis of physical parameters also warrants attention. By identifying which physical constraints exert the greatest influence on model predictions, such analysis can reveal avenues for model simplification and highlight those parameters that demand the most careful experimental characterization, thereby guiding both model development and future data collection efforts [34].

### 4.2. Integration of LCA

#### 4.2.1. Methodological Foundations and Challenges in LCA Integration

Before outlining the integration framework for coupling PIML with life cycle assessment, it is important to recognize several inherent methodological complexities in LCA that pose particular challenges for this combined approach [45]. The definition of the functional unit is a foundational issue. The reference against which performance is measured, whether per cubic meter of treated water, per kilogram of pollutant removed, or per year of operation, critically shapes the conclusions drawn when comparing different scenarios and technologies. For biochar-based systems, this choice becomes especially intricate because biochar serves multiple functions simultaneously: it not only treats water but can also act as a soil amendment and contribute to carbon sequestration [46]. These cross-domain benefits must be somehow reconciled within a single functional unit, which is far from straightforward. Equally consequential is the selection of system boundaries. Deciding where to draw the line around the system under study is a critical LCA decision. For biochar, a wide array of stages may be considered for inclusion, including feedstock cultivation, transportation, pyrolysis, chemical modification, water treatment application, regeneration cycles, and ultimate disposal or beneficial reuse. Each inclusion or exclusion of upstream and downstream processes can shift the overall results in nontrivial ways, making boundary selection a source of significant variability across studies. Allocation procedures introduce another layer of complexity. When biochar production yields co-products such as bio-oil and syngas, the environmental burdens associated with the production process must be distributed among these multiple outputs [47]. The allocation basis selected, whether derived from mass, energy, or economic value, can substantially alter the final conclusions of an LCA [45]. Transparent justification of the selected approach is therefore essential, yet consensus on a preferred method remains elusive. Regional variability further complicates the picture. LCA results for biochar are highly location-dependent, as factors like electricity grid composition, feedstock availability, local water quality, and regulatory conditions differ enormously across geographies [46]. Finally, data quality and uncertainty present persistent challenges. Life cycle inventory data for novel biochar materials are frequently incomplete or derived from small-scale laboratory operations, which introduces considerable uncertainty [47]. This uncertainty originates in the inventory phase and propagates through the impact assessment to the final results, necessitating systematic methods for tracking and quantifying its influence. If these uncertainties are not rigorously addressed, the reliability of LCA outcomes along with any PIML-LCA coupling derived from them cannot be taken for granted [48].

#### 4.2.2. Limitations in Biochar Research

LCA as a standardized tool for quantifying environmental impacts across a product’s entire life cycle, has been preliminarily applied in biochar research [45]. However, existing studies suffer from several major shortcomings that hinder comprehensive sustainability evaluation. One critical limitation is the severe lack of foundational inventory data. This is particularly evident in the insufficient data on raw material consumption, energy use, and pollutant emissions during the preparation stage of composite materials, making it difficult to accurately characterize the environmental burdens of novel biochar materials [47]. Another shortcoming is the incomplete quantification of environmental impacts. Most studies focus only on limited indicators such as global warming potential and energy consumption, while neglecting other critical impact categories like acidification, eutrophication, human toxicity, and ecotoxicity, leading to an incomplete picture of the true environmental trade-offs [48]. Furthermore, there is an absence of long-term performance and uncertainty assessment. Existing LCA studies fail to consider performance degradation over extended operation periods and multiple regeneration cycles, and therefore cannot quantify the actual environmental benefits throughout the full life cycle of biochar systems [4]. Finally, economic feasibility assessment remains fragmented. Current research typically accounts only for raw material costs, while overlooking key economic factors across the entire supply chain, including equipment investment, operational energy consumption, regeneration costs, labor expenses, and carbon credit revenues [46].

The effective integration of PIML and LCA also requires addressing uncertainty propagation across the coupled system. PIML predictions carry uncertainty from model parameters and data; these uncertainties must be propagated through the LCA module to produce confidence intervals for environmental impact estimates. Monte Carlo sampling is recommended for this purpose, with uncertainty in PIML predictions represented through Bayesian posterior distributions [44]. This approach enables probabilistic assessment of sustainability outcomes rather than single-point estimates, providing more robust decision support.

#### 4.2.3. A Three-Step Framework for Multi-Objective Optimization

A deep integration framework combining PIML and LCA is established to achieve coordinated optimization of material performance, environmental impact, and economic cost. This framework consists of three sequential steps [49]. Using biomass feedstock, preparation parameters, modification methods, and wastewater treatment operating conditions as inputs, the PIML model predicts core performance indicators including pollutant removal efficiency, adsorption capacity, degradation rate, and treatment capacity, as well as operational characteristics such as number of regeneration cycles, performance decay rate, service life, and difficulty of solid–liquid separation. Based on PIML predictions and following LCA standards, comprehensive environmental and economic indicators are calculated across the entire life cycle [45]. Environmental indicators include global warming potential, acidification potential, eutrophication potential, human toxicity potential, water consumption, and energy consumption. Economic indicators comprise production cost, regeneration cost, disposal cost, carbon credit revenue, unit pollutant treatment cost, and payback period [46]. Feedstock type, pyrolysis parameters, modification processes, and operating conditions are used as decision variables. The optimization objectives are to maximize pollutant removal efficiency, minimize environmental footprint, and minimize treatment cost, while compliance with emission standards, material stability, and energy consumption limits serve as constraints. Pareto-optimal solutions are obtained through multi-objective optimization algorithms such as Non-dominated Sorting Genetic Algorithm II (NSGA-II) and Multi-Objective Evolutionary Algorithm based on Decomposition (MOEA/D), providing decision-making strategies for sustainable biochar materials and process design [49].

#### 4.2.4. Unit Treatment Cost Estimation Model

Based on the biochar life cycle cost model, combined with PIML prediction results, the unit pollutant treatment cost can be accurately quantified. The unit treatment cost, expressed in USD per kilogram of pollutant treated, is calculated using the following equation [46]:

(6) $$C_{unit}=\frac{C_{prod}+\left(C_{reg}\times N_{reg}\right)+C_{disp}-B_{env}}{M_{total}}$$

where C_unit_ is the unit treatment cost (USD per kg pollutant removed), C_prod_ is the production cost of biochar per unit mass (USD per ton biochar), C_reg_ is the single regeneration cost per unit mass (USD per ton biochar per cycle), N_reg_ is the effective number of regeneration cycles (dimensionless), C_disp_ is the waste disposal cost per unit mass (USD per ton biochar), B_env_ is the environmental benefits (e.g., carbon credits, soil improvement gains) per unit mass (USD per ton biochar), and M_total_ is the total mass of pollutants treated per unit mass of biochar (kg pollutant per ton biochar).

The total mass of pollutants treated, M_total_ can be further expressed in terms of the initial adsorption capacity [4]:

(7) $$M_{total}=q_{0}\times (N_{reg}+1)$$

where $q_{0}$ is the initial adsorption capacity (kg pollutant per ton biochar).

Substituting this expression, the unit treatment cost (Equation (6)) can also be rewritten as

(8) $$C_{unit}=\frac{C_{prod}+\left(C_{reg}\times N_{reg}\right)+C_{disp}-B_{env}}{q_{0}\times (N_{reg}+1)}$$

This formulation integrates life cycle costs and benefits to accurately assess the economic feasibility of different processes [46].

#### 4.2.5. Illustrative Case Study: Heavy Metal Removal with Layered Double Hydroxides—Biochar Composite

To illustrate the practical utility of the proposed PIML-LCA integration framework, we present a hypothetical case study based on published data for a ZnAl-layered double hydroxide modified biochar for Pb^2+^ removal from electroplating wastewater [17]. Using literature-reported parameters (pyrolysis temperature: 600 °C, ZnAl loading: 10% *w*/*w*, initial Pb^2+^ concentration: 100 mg/L, adsorbent dosage: 1 g/L, pH 5.5), we demonstrate the workflow:

(i) PIML prediction: A physics-informed neural network embedding Langmuir isotherm q_e_ = q_m_K_L_C_e_/(1 + K_L_C_e_) and pseudo-second-order kinetics dq_t_/dt = k_2_(q_e_ − q_t_)^2^ would predict equilibrium adsorption capacity (q_m_ ≈ 180 mg/g, K_L_ ≈ 0.15 L/mg) and rate constant (k_2_ ≈ 0.008 g/mg·min).
(ii) LCA-based performance estimation: Using the cost model from Section 4.2.4, with the following parameter values adopted from literature [6,11]: C_prod_ = $850/ton [6], C_reg_ = $120/cycle, N_reg_ = 5 effective regeneration cycles, C_disp_ = 50 USD/ton biochar, B_env_ = 45 USD/ton biochar (carbon credit revenue), and $q_{0}$ = 180 kg/ton biochar [17], the unit treatment cost is estimated as:
  C_unit_ = [850 + (120 × 5) + 50 – 45]/[180 × (5 + 1)] ≈ $1.35 per kg Pb^2+^ removed
(iii) Multi-objective optimization: Varying pyrolysis temperature (400–800 °C) and layered double hydroxides loading (5–20% *w*/*w*) within the PIML-LCA framework would identify Pareto-optimal solutions balancing adsorption capacity, energy consumption, and treatment cost [49]. For instance, increasing pyrolysis temperature from 600 °C to 750 °C may increase q_m_ by 15% but increase energy consumption by 30%, revealing trade-offs that require careful optimization.

The robustness of the unit treatment cost estimate was evaluated by varying key parameters within plausible ranges reported in the literature (±20% for production cost, ±30% for regeneration cost, ±2 cycles for regeneration number). Results indicate that the unit treatment cost ranges from approximately $1.18 to $1.85 per kg Pb^2+^ removed, with production cost and regeneration cycles identified as the most influential parameters [48]. This sensitivity range underscores the importance of accurate cost data and suggests that efforts to reduce production costs or extend biochar service life would yield the greatest economic benefits.

This illustrative example demonstrates how the PIML-LCA framework enables rapid screening of design parameters and quantitative sustainability assessment, guiding experimental efforts toward economically and environmentally optimal solutions. While this case study is hypothetical, it is grounded in real literature parameters and illustrates the practical decision-support capability of the proposed framework.

### 4.3. Establishing a Water–Energy–Soil–Food Closed-Loop System

#### 4.3.1. Description of System Components

**Figure 2:**
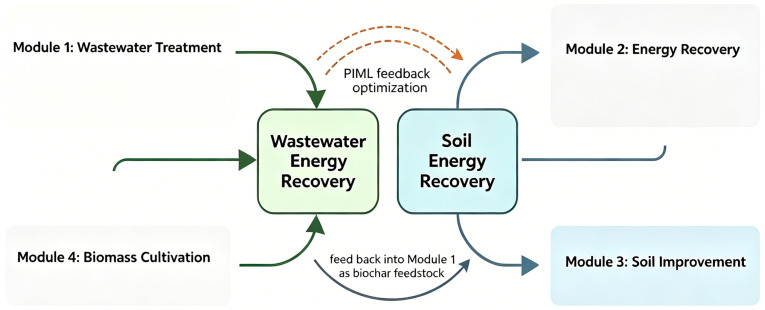
Conceptual diagram of the water–energy–soil–food closed-loop system for sustainable biochar application. This circular system comprises four interconnected components. Component 1: Wastewater treatment, where biochar-based adsorption and catalytic degradation remove heavy metals and organic pollutants, while nutrients (nitrogen and phosphorus) become concentrated on the biochar surface. Component 2: Energy recovery, in which spent biochar undergoes gasification or pyrolysis to recover combustible gases, bio-oil, and bio-hydrogen, generating electricity or heat to achieve energy self-sufficiency. Component 3: Soil improvement, where residual biochar enriched with organic carbon, nitrogen, and phosphorus is applied as a slow-release carbon-based fertilizer. Component 4: Biomass cultivation, in which improved soil supports the growth of energy crops, fiber crops, and food crops, thereby providing feedstock for biochar production and closing the loop. Arrows indicate material flow direction. Feedback loops (dashed arrows) represent PIML-guided optimization and real-time control.

Breaking through the limitations of single water treatment units, we construct a water–energy–soil–food resource recycling closed-loop system, enabling synergistic development in pollutant resource recovery, energy self-sufficiency, soil improvement, and food production (Figure 2) [50]. In the wastewater treatment component, biochar serves as the core material, efficiently adsorbing and catalytically degrading heavy metals and organic pollutants in water, while simultaneously concentrating nutrients such as nitrogen and phosphorus from wastewater, ensuring that treated effluent meets discharge standards [5]. For energy recovery, spent biochar loaded with biomass residues and organics undergoes gasification or pyrolysis to recover combustible gases, bio-oil, and bio-hydrogen, generating electricity or heat to meet the system’s own energy demands and achieving energy self-sufficiency [51]. In terms of soil improvement, the residual biochar after energy recovery, which is rich in organic carbon, nitrogen, and phosphorus, is applied to farmland as a slow-release carbon-based fertilizer, enhancing soil organic matter content, water and nutrient retention, as well as carbon sequestration and emission reduction, thereby generating carbon credit revenues [46]. Finally, biomass cultivation is supported by the improved soil, which promotes the growth of energy crops, fiber crops, and food crops. The harvested biomass is then used as feedstock for biochar production, returning to the wastewater treatment stage and completing the closed loop [50]. This system fully realizes the transformation of waste into valuable resources, converting wastewater pollutants and discarded biomass into water, energy, soil nutrients, and food, thus maximizing environmental, economic, and social benefits.

#### 4.3.2. Role of PIML in the Closed-Loop System

PIML acts as the intelligent brain of the closed-loop system, guiding decision-making and control throughout the entire process [9]. For node performance prediction, PIML accurately forecasts outputs across all stages, including wastewater treatment efficiency, nutrient recovery rate, energy output, crop yield, and carbon sequestration. In terms of multi-objective co-optimization, PIML balances water treatment performance, energy recovery efficiency, soil improvement outcomes, and crop productivity to achieve optimal system operation [49]. For dynamic real-time control, based on real-time monitoring data of water quality, soil conditions, and energy status, PIML dynamically adjusts preparation parameters, operating conditions, and operational strategies to adapt to fluctuating conditions. Finally, for lifecycle assessment, PIML continuously quantifies the environmental, economic, and carbon reduction benefits of the closed-loop system, informing iterative upgrades and improvements [48].

#### 4.3.3. Critical Risk Assessment and Implementation Barriers

The water–energy–soil–food closed-loop system, while conceptually attractive, faces several critical risks and implementation barriers that must be rigorously addressed before practical deployment [50]. The most significant concern is contaminant accumulation in recovered products. Biochar used for wastewater treatment inevitably adsorbs or concentrates heavy metals (e.g., Pb^2+^, Cd^2+^, Cr^6+^), persistent organic pollutants, and pathogens. When spent biochar is subsequently gasified for energy recovery, some contaminants may volatilize, requiring costly emission controls; when applied to soil, they may leach into groundwater or be taken up by crops, creating food safety risks [52]. This creates a contaminant treadmill where pollutants simply cycle through the system rather than being permanently sequestered or destroyed.

Beyond the primary challenges, four secondary concerns warrant attention. During repeated adsorption–regeneration cycles, biochar undergoes surface oxidation, pore clogging, active site loss, and metal nanoparticle aggregation [4]. These progressive changes reduce adsorption capacity, degrade catalytic activity, and compromise treatment performance, undermining the cost-effectiveness of regeneration cycles and requiring realistic assessment of service life and economic viability. While biochar can concentrate nitrogen and phosphorus from wastewater, their bioavailability to crops is often limited. Nitrogen is primarily recovered as ammonium (NH_4_^+^) rather than plant-available nitrate, and phosphorus may be bound in recalcitrant forms [50]. Moreover, competition between nutrients and heavy metals for limited adsorption sites reduces overall recovery efficiency. In most jurisdictions, land application of materials derived from wastewater treatment is subject to stringent regulations (e.g., US EPA Part 503 biosolids rules, EU Fertilizer Products Regulations). Spent biochar may not qualify as a soil amendment without extensive safety testing and certification, and regulatory approval can be time-consuming and costly, potentially preventing the “soil improvement” component in practice [47]. The cumulative capital and operational costs of additional system components (regeneration units, gasification equipment, soil application logistics) are substantial. Current carbon credit prices ($40–80/ton CO_2_-eq) may not fully offset these costs [46].

Mitigation strategies to address these risks include: (a) contaminant-specific post-treatment, such as acid washing to remove heavy metals before soil application; (b) hybrid disposal pathways, where contaminant-laden biochar is sent to hazardous waste landfills or vitrified for secure disposal rather than land-applied; (c) life-cycle toxicity assessment integrated into the LCA framework [48]; (d) policy and regulatory engagement to, develop risk-based standards; and (e) adaptive management, where real-time monitoring and PIML-based decision support dynamically route spent biochar to the safest end-use based on contaminant loading and market conditions [9]. Additionally, long-term environmental risks such as slow desorption of organic pollutants, mobilization of metals under changing pH conditions, and biochar degradation products (e.g., polycyclic aromatic hydrocarbons, nanoparticles) remain poorly understood and may introduce chronic ecological and human health risks [52].

These considerations do not invalidate the closed-loop vision but underscore that its implementation requires: (i) contaminant-specific management strategies (e.g., selective use of biochar for low-contaminant wastewaters, acid washing); (ii) performance monitoring and predictive maintenance; (iii) regulatory engagement and risk-based standard development; and (iv) economic analysis including sensitivity to carbon prices and technology costs [46]. PIML can contribute by predicting contaminant accumulation patterns, optimizing regeneration schedules, and supporting real-time decision-making for safe biochar routing. Careful system design, rigorous safety testing, and appropriate regulatory frameworks are essential for responsible deployment.

## 5. Research Roadmap

### 5.1. Data Infrastructure and Physical Model Development

This phase is to establish a high-quality, standardized, and structured database for biochar-based water treatment along with a knowledge base of physical models, thereby laying the technical foundation [53]. Key tasks in this phase include building a six-dimensional standardized database that covers feedstock, process, characterization, performance, environment, and economics, while defining unified data schemas and quality grading criteria [54]. Another important task is to systematically organize, encode, and modularize physical models for biochar adsorption, degradation, mass transfer, and reactor dynamics, forming a callable library of physical knowledge modules [10]. In addition, global open literature data and in-house experimental data are integrated, followed by data cleaning, standardization, and annotation to build a high-quality dataset [36]. Baseline traditional machine learning models such as random forest, gradient boosting decision trees, and artificial neural networks are also developed, and performance benchmark testing is conducted [6]. The expected outputs of this phase include a publicly accessible standardized biochar wastewater treatment database, a Python toolkit for physical knowledge, and a baseline model performance report.

### 5.2. PIML Model Development and Validation

The core objective of this phase is to develop PIML models that are robust with small datasets, highly interpretable, and strongly generalizable, and to validate them across multiple scenarios [9]. Key tasks involve developing core PIML algorithms including physics-informed neural networks, physics-constrained ensemble learning, and Bayesian physical inference [10]. The models are then trained and validated for five major pollutant categories, namely heavy metals, antibiotics, dyes, polycyclic aromatic hydrocarbons, and endocrine-disrupting compounds [5]. Robustness tests with limited data, cross-scale generalization tests, and physical interpretability evaluations are conducted to optimize model performance [34]. Furthermore, PIML is integrated with LCA models, and a multi-objective optimization module is developed [49]. The expected outputs of this phase include an open-source PIML toolbox, and validation reports for multi-pollutant scenarios.

### 5.3. System Integration and Engineering Validation

It is important to acknowledge that, to date, publicly available case studies of PIML-driven biochar water treatment at pilot or industrial scale are essentially absent [55]. The challenges of industrial adoption extend beyond technical modeling: they include industry inertia, proprietary data concerns, process engineers’ unfamiliarity with PIML methodologies, and regulatory requirements for validation using approved conventional approaches [47]. The pathways described in this section are therefore forward-looking proposals rather than descriptions of established practices. We emphasize that the transition from laboratory PIML models to industrial applications requires systematic pilot-scale demonstration, iterative model refinement, and regulatory engagement. This process is inherently protracted, and even under the most favourable scenarios, it is realistic to anticipate a timeline of five to ten years [55]. Recognizing the current absence of large-scale industrial PIML implementations, we propose a graduated validation approach that begins with laboratory-scale benchmarking in the first two years, during which PIML models are validated against comprehensive laboratory datasets, including multi-component pollution scenarios and variable operating conditions [9]. This is followed by pilot-scale demonstration from year three to year five, in collaboration with industrial partners, to install PIML-assisted monitoring and control at pilot-scale treatment facilities (e.g., 1–10 m^3^/day), enabling model validation under realistic yet controllable conditions [55]. The final stage involves full-scale deployment from year five to year ten, scaling up to industrial treatment plants (100–10,000 m^3^/day) with continuous model refinement and integration with plant control systems [43]. This phased approach mitigates risk and allows iterative learning that enhances both model reliability and stakeholder confidence.

This phase aims to achieve integration of the water–energy–soil–food closed-loop system and conduct pilot-scale and industrial-scale engineering validation [50]. Key tasks include developing a visualization simulation platform for the closed-loop system that integrates PIML, LCA, and multi-objective optimization modules [49]. Pilot-scale validation is conducted in three typical scenarios, namely electroplating heavy metal wastewater, textile dyeing organic wastewater, and pharmaceutical antibiotic wastewater [5]. Industrial operational data are then collected to enhance cross-scale generalization capability of the mode and achieve engineering adaptation [38]. A sustainable design guideline for biochar-based wastewater treatment is also developed, along with draft industry standard proposals to promote technology dissemination [47]. The expected outputs of this phase include a closed-loop simulation platform, pilot and industrial validation reports, a sustainable design guideline, and draft industry standards.

Traditional statistical interpretability tools can provide correlation-based explanations between features and outputs, failing to meet the causal explanation requirements for biochar design [7]. Within a PIML framework, a dual-dimensional explainability system integrating physics and statistics must be established to achieve not only descriptive understanding but also causal explanation [9]. This system comprises three layers of tools: the first layer consists of physical consistency verification tools, which validate whether model predictions align with adsorption thermodynamics, kinetics, and mass transfer principles [10].

### 5.4. Prioritized Milestones and Critical Dependencies

To guide research investment and effort prioritization, we distinguish short-term (1–2 years), medium-term (3–5 years), and long-term (5–10 years) objectives, while recognizing that each phase is critically dependent on foundational progress and external partnerships [55]. In the short term, efforts must focus on establishing a standardized, publicly accessible biochar database with consistent data schemas [54], alongside the development and validation of baseline PIML models for single-pollutant adsorption using existing literature data [9]. This initial phase involves benchmarking PIML performance against conventional machine learning approaches across diverse biochar systems [6]. It also includes publishing open-source PIML toolboxes for adsorption prediction, alongside defining physical consistency metrics and validation protocols [10]. Achieving these goals depends on adequate funding for database infrastructure, active community engagement for data sharing, and the availability of comprehensive literature datasets [53].

In the medium term, over the next three to five years, the scope expands to extending PIML models to multi-pollutant systems and complex water matrices [5], developing integrated PIML-LCA frameworks with uncertainty quantification [48], and conducting pilot-scale validation in collaboration with industry partners [55], while also implementing transfer learning and active learning strategies for cross-scale generalization and establishing benchmark datasets for model comparison [9]. These medium-term advances, however, are contingent upon securing industrial pilot partnerships, investment in monitoring infrastructure, and the development of robust domain adaptation algorithms. Looking further ahead to the five- to ten-year horizon, long-term priorities envision full-scale industrial deployment with real-time PIML-assisted process control, the implementation of water–energy–soil–food closed-loop systems in suitable contexts, the development of industry standards and regulatory frameworks for PIML-guided biochar treatment, and the quantification and monetization of environmental, economic, and social benefits at system scale [43,46,47,50]. Achieving these long-term goals will depend critically on regulatory evolution, the development of carbon credit markets, successful pilot-scale outcomes, and sustained improvements in economic viability. Overall, this phased prioritization underscores that successful PIML implementation is inherently contingent on foundational data infrastructure that is currently lacking; without addressing data standardization and sharing, subsequent modeling advances will be severely constrained, and industrial-scale validation cannot proceed without robust partnerships and data-sharing arrangements [53]. By highlighting these dependencies, we aim to inform realistic expectations and guide strategic research planning accordingly.

## 6. Conclusions and Outlook

Biochar-based wastewater treatment technology is at a critical juncture where thepotential for transitioning from laboratory-based empirical research to intelligent industrial applications is increasingly recognized. However, realizing this potential requires overcoming substantial scientific and engineering challenges. This perspective presents a conceptual roadmap toward this transition, identifying key enabling technologies and critical knowledge gaps. The dual limitations of traditional trial-and-error approaches and purely data-driven black-box models represent the main challenges hindering progress in this field. This study proposes a novel paradigm that deeply integrates PIML with LCA [48]. By incorporating physical knowledge constraints, this paradigm addresses challenges related to small sample sizes, interpretability, and cross-scale generalization. Through LCA, it enables coordinated optimization across environmental, economic, and energy objectives. By reconstructing resource circulation via the water–energy–soil–food closed-loop system, it provides a new pathway toward efficient, intelligent, sustainable, and scalable deployment of biochar technologies [50].

The core value of this paradigm lies in three dimensions. On the scientific front, data-driven methods are unified with physical laws, making predictions that are not only statistically reliable but also physically consistent. At the systematic level, a shift occurs from single-pollutant removal toward holistic optimization across the entire life cycle and resource flows, thereby breaking through the boundaries of conventional technologies [49]. When viewed from a practical standpoint, this paradigm bridges the gap between laboratory research and industrial implementation, offering actionable tools for large-scale application of biochar wastewater treatment [55]. With deep interdisciplinary integration among environmental science, artificial intelligence, systems engineering, and agricultural science, along with continuous improvements in data infrastructure, iterative advancements in PIML algorithms, and successful engineering deployment of closed-loop systems, biochar wastewater treatment will overcome current limitations and emerge as a cornerstone technology for water pollution control, resource recycling, carbon reduction, and sustainable agriculture. It will thus play a pivotal role in achieving carbon neutrality goals and advancing ecological civilization.

## Figures and Tables

**Table 1:** Summary of common machine learning algorithms, their core applications, and optimal predictive performance (R^2^) in biochar water treatment research [28,29,30,31,32,33].

Machine Learning Algorithm	Core Applications	Optimal Performance Metric (R^2^)
Random forest	Prediction of biochar yield/carbon content, fitting of adsorption capacity, forecasting of pollutant removal efficiency	0.9873
Artificial neural network	Adsorption kinetics modeling, optimization of preparation parameters, prediction of multi-pollutant adsorption	0.9992
Gradient boosting decision tree	Photocatalytic degradation modeling, prediction of pollutant degradation rate	0.9840
Support vector machine	Fitting of adsorption data with small samples, prediction of treatment performance under complex water quality conditions	0.9986
CatBoost	Prediction of emerging pollutant adsorption capacity, analysis of synergistic effects of multiple factors	0.9433
Decision tree	Screening of key influencing factors, determination of threshold values for preparation parameters	0.8542

## Data Availability

There are no new data.
